# Decolorization of methyl orange and aniline red dyes by *Enterococcus hirae* isolated from beach sand

**DOI:** 10.1038/s41598-025-12584-5

**Published:** 2025-09-29

**Authors:** Ganiveth Manjarrez-Paba, Alfredo Montes-Robledo, Henry Cuevas-Menco, Dayana Baena-Baldiris, Rosa Baldiris Avila

**Affiliations:** 1 Grupo de Investigación Microbiología Clínica y Ambiental, Facultad de Ciencias Exactas y Naturales, Universidad de Cartagena, Cartagena, Colombia; 2 Doctorado en Ciencias, Facultad de Ciencias Exactas y Naturales, Universidad de Cartagena, Cartagena, Colombia; 3 Profesor, Facultad de Ciencias de la Salud, Universidad del Sinú Seccional Cartagena, Cartagena, Colombia

**Keywords:** Aniline red, Methyl orange, Decolorization, *Enterococcus*, Dyes, Environmental microbiology, Bacteria

## Abstract

Azo dyes are categorized as toxigenic colorants, and the untreated discharge of effluents containing these compounds poses serious risks to both the environment and human health. Therefore, the objective of this study was to isolate and characterize an *Enterococcus hirae* Alf123 strain, obtained from beach sand, with the ability to degrade the azo dyes methyl orange and aniline red, to evaluate its decolorization capacity, and to propose a possible degradation pathway for both dyes. The bacterial strain was isolated from beach sand samples using selective culture media, and its identity was confirmed through biochemical tests, *tuf* gene amplification, and 16S rRNA gene sequencing. Decolorization assays were carried out using various dye concentrations. In addition, the activity of several enzymes laccases, lignin peroxidase, manganese peroxidase, and dioxygenases was evaluated, and spectroscopic analyses (UV–Vis and FT-IR) were employed to monitor structural changes in the dyes during degradation. The Alf123 strain achieved complete decolorization of methyl orange and aniline red at concentrations up to 200 mg/L and maintained activity at concentrations as high as 500 mg/L. Lignin peroxidase was the most efficient enzyme, reaching decolorization rates of 47.43% for methyl orange and 33.98% for aniline red, followed by manganese peroxidase with rates of 27.38% and 20.02%, respectively. Based on experimental results and literature review, a possible degradation mechanism and metabolic pathway used by *E. hirae* Alf123 strain for both dyes were proposed.

**Supplementary Information:**

The online version contains supplementary material available at 10.1038/s41598-025-12584-5.

## Introduction

The synthetic dyes are used in multiple industrial activities, including textiles, food, pharmaceuticals, paper, leather, and cosmetics. This is due to their wide range of colors, ease of production, and low cost^1,2^. Synthetic dyes are classified based on their chemical structure, where the functional groups responsible for color, known as chromophores, include azo (-N = N-), carbonyl (-C = O), nitro (-NO_2_), methine (-CH =), and quinoid groups. Additionally, auxochromes, such as amines (-NH_3_), hydroxyls (-OH), carboxyls (-COOH), and sulfonates (-SO_3_H), modify the intensity and tone of the color^3,4^.

Among synthetic dyes, azo dyes represent the most significant class, accounting for 60% to 70% of those used in the textile and dyeing industries^5^. These dyes are characterized by the presence of one or more azo (-N = N-) bonds connected to aromatic structures^6^. They have proven highlyuseful in various industries due to their wide range of colors, greater stability against degradation caused by sunlight and rain, and the limitations associated with natural dyes^7^. However, a considerable percentage of these compounds, ranging from 4 to 12%, is lost during production and dyeing processes, resulting in industrial effluents containing traces of untreated dyes^8^.

Azo dyes, widely used across industries, have demonstrated high toxicity, posing significant risks to human health, aquatic and terrestrial ecosystems, and triggering environmental imbalances^9^. The degradation of an azo dye generally leads to the cleavage of the chromophore and the disappearance of its color. Once azo dyes are degraded using physicochemical and biological techniques to remove the color (decolorize) from dye-containing wastewater^10^, a significant amount of aromatic amine compounds can be generated. Aromatic amine compounds, especially aniline, often emerge and appear in wastewater during the degradation processes of azo dyes^11–13^. These toxic intermediates could threaten various living organisms (aquatic and terrestrial) and humans and pose long-term risks to different ecosystems^1,14^.

**Fig. 1:**
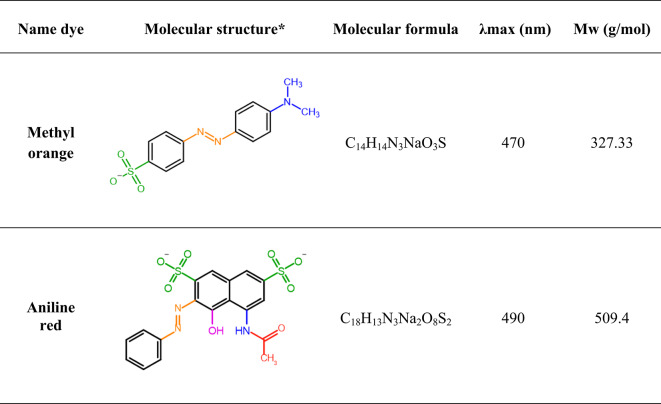
Chemical structure and characterization of aniline red and methyl orange dyes. *Red: Chromophore group, blue and purple: auxochrome group and green: water soluble group.

Methyl orange is a synthetic monoazo organic dye derived from benzene, sulfanilic acid, and dimethylaniline (Fig. 1). It is a water-soluble anionic heterocyclic sulfonated dye widely used in various industries, such as paper manufacturing, and as a pH indicator due to its ability to change color depending on the medium’s acidity or alkalinity^15^. In some regions, it is also used as a food colorant, although its toxicity at high concentrations has led to restrictions in certain areas^16^. Methyl orange belongs to the azo dye group and poses environmental risks. It can cause soil infertility by increasing salinity, reducing crop yields, and affecting biodiversity, particularly flora^17^.

Aniline red is an organic compound and chemically represents a monoazo dye that contains benzene and naphthyl groups, it also has two sulfonate groups, and an amide group attached to an aromatic ring. (Fig. 1)^18^. Aniline plays a crucial role in the synthesis of Aniline red. Through a diazotization reaction, aniline is converted into a diazonium salt, which subsequently undergoes coupling with a naphthol derivative to form the characteristic azo bond (-N = N-). This process results in the formation of Aniline red, a vibrant dye with strong chemical stability and solubility due to the presence of sulfonate groups^19^.

Aniline is widely used as a raw material in dye production and is also a vital precursor in synthesizing other chemicals such as dyes, pesticides, explosives, plastics, pharmaceuticals and herbicides^20^. Its primary chemical function lies in acting as an intermediate in chemical reactions due to its nucleophilic capacity to form bonds through electrophilic substitution. It is also formed during the degradation of numerous azo dyes, thus requiring additional treatment before disposal^21,22^. This precursor and dye intermediate poses an environmental challenge due to its toxicity and persistence in the environment^23^. This compound can easily bind to colloidal organic materials and is difficult to degrade naturally, once released into the environment aniline alters the physicochemical properties of water, affects biological populations, and poses risks to aquatic ecosystems^24^.

Despite the existence of specific regulations aimed at ensuring the proper treatment of effluents contaminated with dyes and their derivatives, non-compliance by certain organizations remains a significant obstacle to addressing environmental pollution caused by these toxic products^25^. The release of these compounds without proper pretreatment not only violates environmental laws but also threatens biodiversity^26^. Aquatic ecosystems are particularly affected as dyes alter water quality and photosynthesis, while terrestrial ecosystems suffer from soil contamination and reduced fertility^27,28^. Therefore, it is crucial to develop sustainable alternatives for the treatment of industrial effluents to preserve ecosystem sustainability and mitigate environmental damage^29^. Although chemical and physical treatments have been widely used, they often generate secondary problems, such as the accumulation of large volumes of salts, residual pollutants, and high operational costs^30,31^. In comparison, biological processes, particularly bioremediation, emerge as a more environmentally friendly alternative for removing dyes from effluents^32^.

Bioremediation, which relies on the action of microorganisms such as bacteria and fungi to improve contaminated environments, has demonstrated great potential for the decolorization and degradation of industrial dyes^33,34^. Bacteria, due to their enzymatic capabilities and genetic adaptations, act as natural bioremediators by efficiently breaking down dyes and reducing their environmental impact^35^. Among these, bacterial strains capable of adapting to adverse conditions, such as the presence of heavy metals and high salt concentrations commonly found in industrial effluents, stand out^36,37^.

Nevertheless, high levels of salinity, toxic metals, and other environmental factors can limit the effectiveness of native bacterial strains by restricting their growth and metabolic activity^38^. Therefore, it is essential to select bacteria resistant or tolerant to these extreme conditions. Recent studies suggest that bacteria isolated from coastal environments exhibit unique characteristics, such as tolerance to environmental stress, pH fluctuations, hypersalinity, and resistance to heavy metals^39^. These bacteria have developed adaptive mechanisms due to the extreme conditions in these transitional zones, where terrestrial, freshwater, and marine ecosystems converge, creating dynamic environmental gradients^40^. The present study focused on isolating and characterizing a bacterium capable of degrading the methyl orange and aniline red azo dye, evaluating its decolorization capacity, and suggesting the possible degradation pathway of both dyes.

## Materials and methods

### Sample collection and isolation of presumptive *Enterococcus* strains

Sand samples were collected from Bocagrande Beach, located in Cartagena de Indias, Colombia. At each sampling point, three representative samples were taken from different zones of the beach (intertidal zone, active zone, and resting zone), at a depth of 10 cm. The samples were stored in airtight bags and kept refrigerated at 4 °C during transport to the Microbiology Laboratory of the San Pablo campus of the University of Cartagena, Colombia, for immediate processing and analysis.

For microbial isolation, 10 g of beach sand were mixed by agitation for 5 min in an Erlenmeyer flask containing 20 mL of Brain Heart Infusion (BHI) broth. The mixture was then left to settle for 15 min. Subsequently, 100 µL of the supernatant (avoiding the sediment) was transferred to plates containing Slanetz and Bartley agar, a selective medium specific for the *Enterococcus* genus^41^. The presumptive *Enterococcus* colonies were recultivated on Slanetz agar and BHI agar supplemented with blood for further identification and characterization.

### Characterization and identification of *Enterococcus* strains

The bacterial isolates exhibiting characteristic colony morphology were subjected to Gram staining and standard biochemical tests to confirm their genus. These tests included growth in 6.5% NaCl, catalase production, growth at 45 °C, and the production of pyrrolidonyl arylamidase. For species-level identification, additional tests were conducted, including carbohydrate fermentation (mannitol, arabinose, xylose, raffinose, lactose, and sorbitol), utilization of 1% pyruvate, pigment production, motility, and arginine decarboxylation^42^. The genus confirmation was accomplished through the amplification by PCR of the *tuf* gene, using a 112 bp amplicon specific to the *Enterococcus* genus. The primers used were F: 5´ TACTGACAAACCATTCATGATG 3´ and R: 5´ AACTTCGTCACCAACGCGAAC 3´^43,44^.

### Identification of the strain using MALDI-TOF mass spectrometry

The identification of the bacterial strain was performed using Matrix-Assisted Laser Desorption/Ionization Time-of-Flight (MALDI-TOF) mass spectrometry^45^. Initially, ribosomal protein extraction was carried out. The bacterial culture was grown in Lysogenic Broth (LB) for 18 h. Subsequently, 500 µL of the culture was centrifuged, and the resulting pellet was suspended in 1 mL of double-sterilized Milli-Q water and centrifuged again. The pellet obtained was mixed with 1 mL of 70% ethanol, centrifuged at 13,000 rpm for 5 min, and then resuspended in equal parts of 70% formic acid and acetonitrile. A 1 µL aliquot of the protein extract was deposited onto a polished steel target and air-dried. Each spot was then overlaid with 1 µL of a matrix solution consisting of 2.5 mg of HCCA dissolved in 250 µL of 50% acetonitrile/2.5% trifluoroacetic acid (v/v). Each biological replicate was applied to three spots, and the measurements were acquired in duplicate. The samples were processed using an AutoFlex Speed MALDI-TOF MS instrument (Bruker Daltonics, Germany).

### Genotypic characterization based on 16S rRNA gene analysis

Genomic DNA was extracted using the DNeasy Blood & Tissue Kit (Qiagen) and used for PCR amplification of a 1500 pb 16S rRNA gene fragment with universal primers 27F and 1492R, following^46^. PCR conditions included 35 cycles of 95 °C for 30 s, 58 °C for 30 s, and 72 °C for 1 min, with an initial denaturation at 95 °C for 5 min and a final extension at 72 °C for 10 min. The PCR products were separated on 1.5% agarose gels, stained with ethidium bromide, and visualized under UV light. The purified amplicons were sequenced and analyzed using NCBI (http://www.ncbi.nih.gov/BLAST/) and the EzBioCloud database (https://www.ezbiocloud.net/) to identify the closest homologous sequences. Phylogenetic trees were constructed using the neighbor-joining algorithm and validated with 1,000 bootstrap replicates in MEGA X software.

### Decolorization activity of methyl orange and aniline red

The decolorization activity was evaluated separately for methyl orange and aniline red. Initially, the maximum absorption (λmax) of each dye was determined by scanning light absorption in the visible range (200–800 nm) at 5 nm intervals using a UV–Vis spectrophotometer. Subsequently, standard curves were constructed with solutions of known concentrations to estimate the remaining concentrations of the dyes. The bacterial isolates were cultured in LB broth supplemented with 100 mg/L of aniline red or methyl orange, with each dye evaluated separately. The medium also contained 0.4% glucose and yeast extract, and incubation was carried out for 48 h at 37 °C and pH 7. The optical density of the culture was adjusted to 1.0 ± 0.2. The decolorization capacity was monitored every 24 h using a UV–Vis spectrophotometer. The isolate capable of growing and decolorizing at 100 mg/L of aniline red and methyl orange was selected as a promising candidate for dye decolorization. The decolorization efficiency was calculated according to the method proposed by^27^.

### Dye degradation under optimal conditions

The decolorization capacity of the isolated strains was determined using UV–Vis spectrophotometry with methyl orange and aniline red at a concentration of 100 ppm. To optimize the decolorization process, a single-factor optimization approach was employed to assess the effect of various parameters on the decolorization rate^47^. The pH was evaluated at values of 5.0, 7.0, 9.0, and 11.0, with adjustments made using HCl or NaOH as needed. The salt concentration was adjusted by adding sodium chloride (NaCl) in different proportions to the medium containing the dyes. This medium was supplemented with NaCl at concentrations of 2%, 4%, 6.5%, 8%, and 10% to evaluate the effect of salinity on the decolorization process. The temperature was tested under conditions of 20 °C, 37 °C, 45 °C, and 55 °C to determine its influence on the microbial activity of the isolated strains. In addition, the incubation time was monitored at intervals of 0, 24, 48, 72, 96, and 120 h to identify the optimal time for maximum decolorization. Finally, different dye concentrations (50 ppm, 100 ppm, 200 ppm, 300 ppm, 400 ppm, 500 ppm, 600 ppm, 800 ppm, 900 ppm and 1000 ppm) were tested to investigate the effect of dye concentration on decolorization capacity. During each experiment, the absorbance of dye solutions at the maximum absorption wavelength (λmax) was measured using a UV–Vis spectrophotometer^22,47^. The decolorization efficiency was calculated by comparing the initial and final absorbance values, and the data were analyzed to determine the optimal conditions for maximum decolorization of the dyes. The decolorization efficiency was calculated using the following equation:

1 $$\% Decolorization = \frac{Ao - At}{{Ao}}*100$$

where A_0_ is the absorbance of the dye solution before decolorization and A_t_ is the absorbance of the dyes solution after decolorization.

### Enzymatic activity

#### Preliminary detection of presumed lignocellulolytic activity and via catechol meta-cleavage

Qualitative tests for laccase production were performed on the isolate. A medium was prepared with the following components (g/L): 14 of bacteriological agar, 1 of KH_2_PO_4_, 0.002 of CuSO_4_·5H_2_O, 0.5 of yeast extract, 4 of fructose, and 0.003 of FeSO_4_·7H_2_O. Additionally, 0.02% (w/v) of guaiacol, 0.04% (w/v) of syringaldazine, 0.006% (w/v) of 1α-naphthol, and 0.05% (w/v) of 2,6-dimethoxy-phenol were weighed separately and added to the medium before sterilizing it in an autoclave for 15 min at 121 °C. Fructose (4 g) was also sterilized separately and added to labeled phenolic plates. The plates were incubated at 30 °C until the appearance of an oxidized color halo^48^.

Bacteria were inoculated on lysogenic agar plates supplemented with 100 mg/L of aniline red and incubated at 37 °C for 24 h to allow growth. Subsequently, a catechol solution was prepared in a 100 mM phosphate buffer (pH 7.0) and added to the culture medium. The plates were then incubated at 37 °C for 48 h to evaluate the phenotypic response of the bacteria in the presence of catechol. The color change in bacterial colonies was used as an indicator of the aniline red degradation pathway. Colonies that turned yellow were presumptively identified as producers of 2-hydroxymuconic semialdehyde, an intermediate in the aniline red degradation pathway via catechol meta-cleavage. In contrast, bacteria that developed a brown coloration suggested the use of an alternative aniline red degradation pathway that does involve catechol ortho-cleavage^49^.

#### Enzymatic assay: the activities of lignin peroxidase, laccase, manganese peroxidase, catechol 2,3-dioxygenase and catechol 1,2-dioxygenase

The enzymatic activities were assayed in cell free; the extract was prepared as the enzyme source for enzymatic assays. Bacterial cells were grown in BHI broth at 37 °C for 24 h and then centrifuged at 11,000 rpm for 5 min at 4 °C. The cells were resuspended in cold 50 mM potassium phosphate buffer (pH 7.4) and subjected to sonication at 50% amplitude with 10 pulses of 30 s, with 2-min intervals at 4 °C. The resulting homogenate was centrifuged at 14,000 rpm for 20 min at 4 °C and the supernatant was used as a crude enzyme source.

The activities of lignin peroxidase, laccase, and manganese peroxidase were measured following established protocols. Lignin peroxidase activity was determined by monitoring the formation of propionaldehyde at 300 nm in a 2.5 mL reaction mixture containing 100 mM n-propanol, 250 mM tartaric acid, and 10 mM H_2_O_2_, as described by^50^. Laccase activity was evaluated in a 2 mL reaction mixture containing 10% ABTS in 0.1 M acetate buffer (pH 4.9), measuring the increase in optical density at 420 nm. Finally, manganese peroxidase activity was determined by observing the formation of the Mn^3+^ tartrate complex at 238 nm in a 2 mL reaction mixture containing 20 mM sodium tartrate buffer (pH 4.5), 0.1 mM MnSO_4_, and 200 μL of the enzymatic extract. The reaction was initiated by adding 0.1 mM hydrogen peroxide^51^.

To assess oxygenase activity, a 3.0 mL reaction mixture was prepared, consisting of 2.0 ml of phosphate buffer (pH 7.0), 0.6 mL of 1 mM catechol, 0.2 mL of deionized water, and 0.2 mL of cell lysate. The reaction was carried out at 25 °C and subsequently analyzed using a spectrophotometer to quantify the formation of 2-hydroxymuconic semialdehyde at 375 nm and muconic acid at 260 nm, thereby determining the enzymatic activity of catechol 2,3-dioxygenase and catechol 1,2-dioxygenase, respectively^52^.

### Determination of aniline red degradation rates and NH₄⁺ ion release

Bacterial cells were grown in liquid LB medium at 37 °C for 48 h. Optical density was measured at 600 nm. Subsequently, 1000 µL of the sample was mixed with 1 ml of cold methanol and centrifuged at 14,000 rpm at 4 °C for 10 min. The concentration of NH₄⁺ ions released from aniline red degradation was determined at 630 nm using an indophenol blue reaction^53^.

### Extraction of metabolites formed after dye degradation

To assess the dye degradation by the isolated bacteria, the culture solutions were collected after 48 h and centrifuged. The resulting supernatants were then used for subsequent analysis. The degradation study was divided into three experimental sections: (i) recording the UV–visible absorbance spectrum of the supernatant, (ii) analyzing the infrared spectrum using a Fourier-transform infrared (FTIR) spectrometer, and (iii) conducting a phytotoxicity test.

#### Fourier transform infrared (FT-IR) analysis of dye and degradation metabolites

The dye and its degraded metabolites were analyzed using FTIR by applying them directly onto a diamond crystal for Attenuated Total Reflectance (ATR). The spectra obtained were corrected for background air absorbance. The measurements were taken in the 4000–400 cm^–1^ range, with each sample being recorded 80 times at a resolution of 4.

#### Phytotoxicity assessment of dye and degradation metabolites

The phytotoxicity assay was performed on *Lactuca sativa* (*L. sativa*) as described by de Moraes et al.^54^ with minor modifications. Experimental levels were prepared at 0.1; 1,0; 10.0 and 100 mg/L. The control groups were Milli-Q Water (negative control) and Zinc sulfate heptahydrate (ZnSO_4_ × 7H_2_O) 0.05 M (Positive control). 20 seeds were placed in sterile Petri dishes with filter paper and soaked with 2 mL of solution from each experimental level of the secondary metabolites. Aftershocks were handled in quintuplicate. The plates were incubated at 28 °C for 5 days. Germination rates, shoot length, and root length were measured, and germination inhibition (GI) data were analyzed^54,55^. The results are presented as the mean and standard error (mean ± SEM).

#### Statistical analysis

All experiments were conducted in triplicate, and the results presented represent the mean values. The data were analyzed using one-way analysis of variance (ANOVA) followed by Tukey–Kramer, Dunn and Shapiro–Wilk tests for comparison. Statistical analyses were performed using GraphPad Prism software version 8.0.2. A *p*-value of less than 0.05 was considered statistically significant.

## Results and discussion

### Characterization and identification of *Enterococcus hirae* strain

The strain with the ability to decolorize methyl orange and aniline red was isolated from beach sand samples. Initially, the identification of the strain was performed at the genus level using standard biochemical tests and specific and enriched culture media Table 1. To confirm the genus level, amplification of the *tuf* gene, which encodes the elongation factor Tu (EF-Tu), was carried out. This factor is a protein that binds to GTP and performs a critical role in protein synthesis^56^. Within the bacterial genome of the genus *Enterococcus*, the *tuf* gene is present in most species, confirming the initial genus-level classification.

**Table 1:** Morphological, physiological, and biochemical characteristics of isolate presumptive *Enterococcus* genus strain.

Morphological and cultural characteristics		
Stain		
Stain	Resulted	Characteristics
Gram	Gram positive	Free cocci, well-defined
Cultural characteristics		
Culture Medium	Resulted	Characteristics
Blood Agar	Alpha hemolysis	Silver-colored, shiny, round, smooth, convex, and small colonies
Slanetz Agar	Growth with red colonies	Small, smooth, moist colonies with defined edges
Biochemical tests and utilization of sugars		
Test	Resulted	Characteristics
Mannitol	Positive	Fermenter
Arabinose	Negative	Non- Fermenter
Xylose	Negative	Non- Fermenter
Raffinose	Positive	Fermenter
Lactose	Positive	Fermenter
Sorbitol	Positive	Fermenter
Potassium tellurite reduction	Negative	No enzymatic reaction
Bile esculin	Positive	Fermenter
Pyruvate	Positive	Fermenter
Arginine decarboxylation	Positive	Amine production
Other phenotypic characteristics		
Test	Resulted	Characteristics
Growth in 6.5% NaCl	Positive	Tolerance to high salinity
Catalase	Negative	No enzymatic reaction
Oxidase	Negative	No enzymatic reaction
Growth at 45 °C	Positive	Presence of colonies

To determine the species, the MALDI-TOF technique was initially used, based on the identification of ribosomal proteins analyzed by mass spectrometry. The results showed an average score of 1.999, associated with *Enterococcus hirae*. Subsequently, the species identification was confirmed through the sequencing of the 16S rRNA gene. The obtained sequence was deposited in the NCBI database under the accession code PQ820966.

**Fig. 2:**
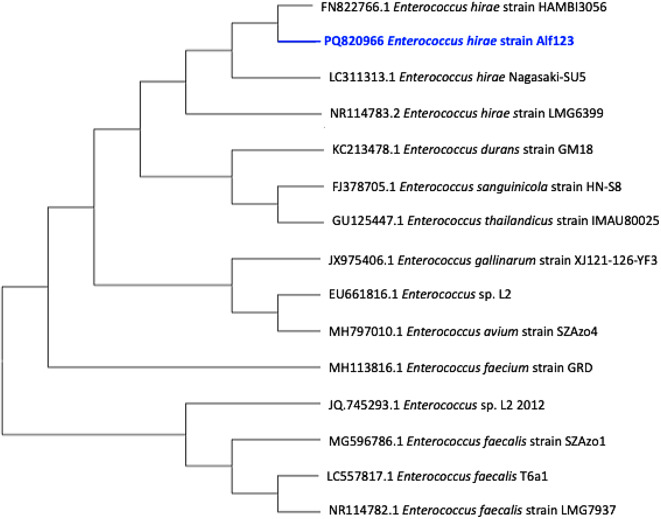
Phylogenetic tree constructed using the 16S rRNA gene sequence of strain Alf123 (*Enterococcus hirae*) generated through the neighbor-joining method.

The phylogenetic analysis of the sequence showed that the strain is closely related to other *Enterococcus* strains with similar genetic lineages, which have been previously reported as useful in bioremediation processes. Several studies have reported the genus *Enterococcus*, including different species such as *E. faecalis*, *E. durans*, *E. sanguinicola*, *E. avium*, and *E. gallinarum*, which have been included in the phylogenetic tree supported by accession codes Fig. 2, with the ability to decolorize synthetic dyes from the azo dye family. However, there are few studies that specifically highlight the species *E. hirae* as a microorganism with this capability. This bacterial genus has demonstrated remarkable tolerance to adverse conditions, such as variations in pH, salinity, and temperature, suggesting its potential application in diverse environments^57^. Moreover, these microorganisms, either in their complete form or using isolated proteins, could metabolize and remove traces of synthetic dyes in effluents, positioning them as viable candidates for scaling up and in situ applications.

### Decolorization activity of methyl orange and aniline red

The decolorization of methyl orange and aniline red dyes was carried out using the Gram-positive bacterial cell *E. hirae Alf123*. The decolorization for the dye samples prior to and after degradation was monitored by observing the spectral peaks in the UV–Vis analysis. The UV–Vis spectrum showed changes in the bacterially treated solutions.

**Fig. 3:**
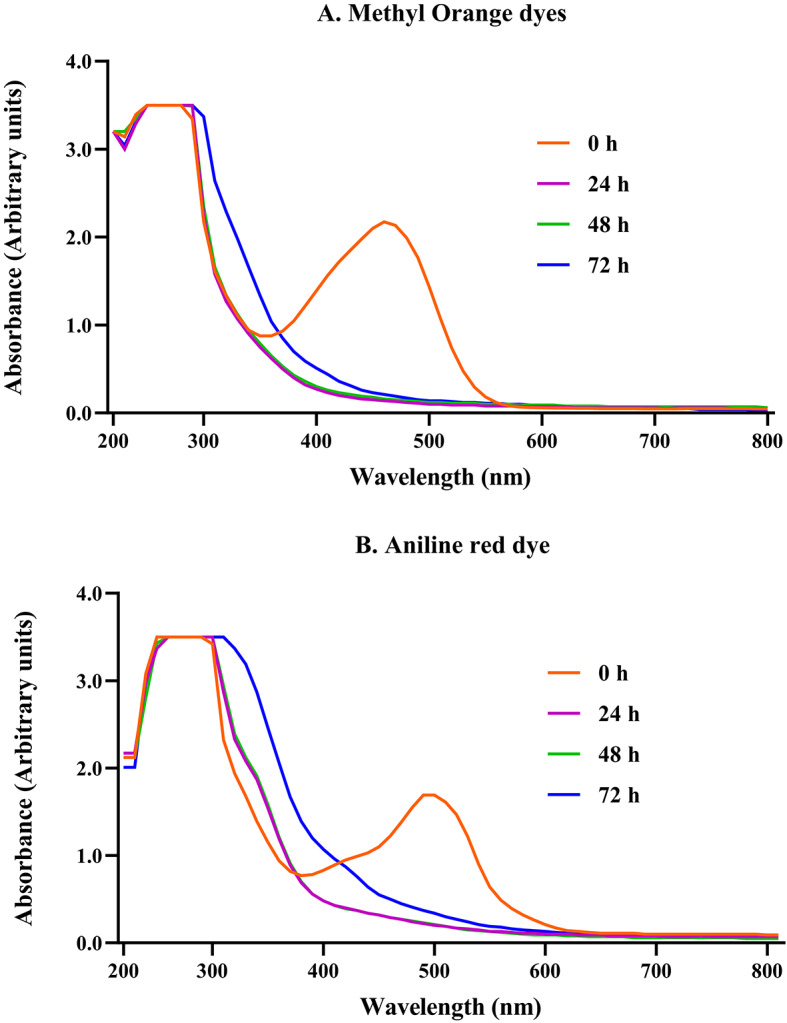
UV visible spectral changes observed during decolorization of methyl orange and aniline red dyes.

During the degradation processes of the methyl orange and aniline red dyes, the decomposition of the chromophore groups was evidenced by the reduction of the initial spectral peaks to wavelengths ʎ = 470 nm and ʎ = 490 nm respectively, visualized in time 0 h (Fig. 3). For Methyl orange, we observed that the absorption peak obtained at 470 nm disappeared after the loss of color orange at 6 h, indicating complete decolorization, by action of the enzyme azoreductase on the “–N = N–” azo bonding. A new absorption peak at 246 nm could be detected after the degradation treatment, attributed at the benzene ring shifts from the position 271 nm. Sulphonated groups were absorbed at 220–230 nm.

In the case of the aniline red, the UV absorption spectra showed two characteristic absorption peaks located at 250 nm and 290 nm attributed to E-band and B-band π-π* electronic transition of the aromatic rings of the dye. Absorption peak at 490 nm was evidenced and is associated with n-π* electronic transitions to the chromophore (azo group (-N = N-), which is responsible for that aniline red appearing colour red strawberry.

After being treated with *E. hirae* Alf 123, we observed that the intensity of the peak in the visible region (490 nm) of dye sharply decreased to almost zero indicating the decolorization with the disruption of the chromophoric group (Azo bond). This change is visible as the solution turns from red strawberry to light yellow. However, the intensities of the two peaks in the UV region (250 nm and 290 nm) do not decrease or disappear after decolorization, suggesting that some undegraded dye was still present in the solution after 72 h of treatment with *E. hirae* Alf123 or by the presence of intermediaries products. Additionally, two new peaks emerged at 320 nm and 410 nm suggesting changes in the aromatic groups.

The changes observed in the absorption peaks before and after decolorization evidence the transformation in the molecular structure of the dye. These results could be attributed in the case of methyl orange, to the azo group (-N = N-) as the main chromophore, along with aromatic groups and the sulfonate group (-SO₃⁻), which act as electronic stabilizers and enhance absorption in the visible region^58^. Characteristic peaks absorbed in the region of 260–300 nm are indicative of potential generation of aromatic amines or phenylazo compounds as intermediaries products of the process of degradation.

In contrast, for aniline red, the absorbance peak at 490 nm was attributed to the azo group (-N = N-) and the aromatic ring, whose electronic interactions contribute to resonance and absorption in the UV region^59^. Absorption peaks at 250 nm and 290 nm were attributed to intermediate results of oxidative or dehydrogenation processes such as naphthalene like molecules, naphthalene sulfonates or derivatives of catechol, as is reported by^60^. A little absorption peak at 320 nm corresponding to the π-π* The bond of N–H observed after 24 h suggests the formation of the benzene amine compound.

While formation of a peak at 420 nm observed after 72 h indicates the formation of possible 3 oxo 4 dienoate. The displacement toward the visible region favors an increase in the rate of degradation. To optimize the efficiency of the decolorization process, various physicochemical parameters were adjusted, including dye concentration, temperature, salt concentration, pH, and exposure time. These adjustments enabled the establishment of an optimal decolorization pattern, maximizing the degradative capacity of the bacterial cells and enhancing the overall process performance.

### Dye degradation under optimal conditions

**Fig. 4:**
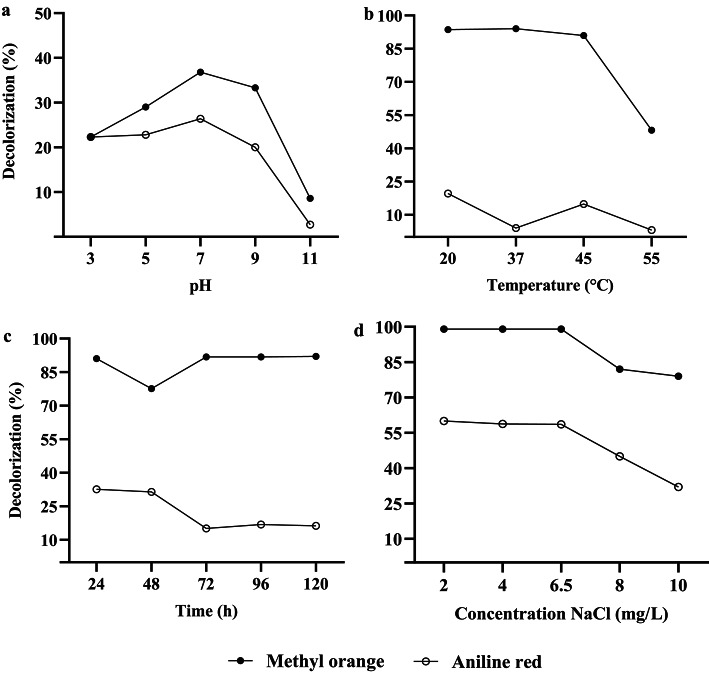
Effect of optimal parameters the decolorization of methyl orange and aniline red. (**a**) Effect of pH. (**b**) Effect of temperature. (**c**) Effect of time incubation. (**d**) Effect of concentration of salt.

The results indicated that the optimal pH for the growth of *E. hirae* in solutions containing methyl orange and aniline red dyes was 7.0 Fig. 4a. While pH preferences may vary, the neutral pH likely promotes electron attraction to functional groups near the azo bond in methyl orange, facilitating its decolorization. Similarly, aniline red showed better decolorization results at neutral pH. This can be attributed to the fact that, although aniline red is not an azo dye, it acts as a precursor. At neutral pH, aniline red predominantly exists in its non-protonated form, which allows the amino group to interact more effectively with cellular components, enhancing the decolorization process. This effect could explain the higher decolorization rate observed at neutral pH^61^. Conversely, as the pH increases, the decolorization rate decreases, possibly due reactions may occur, such as the formation of by-products inhibiting the decolorization reaction^62^. Similarly, at lower pH levels, molecular ionization may interfere with the decolorization process.

Regarding temperature, it is essential to consider the dynamics of the bacterial agent, as *Enterococcus* is a genus of mesophilic bacteria Fig. 4b. At optimal temperatures for these organisms, their metabolic machinery operates efficiently, contributing to a higher rate of dye metabolization^63^. Additionally, these temperatures may positively influence pH stabilization, as hydrogenation at these temperatures could promote the binding of ions electron attraction to functional groups near dyes, facilitating the decolorization process.

Concerning incubation time, it was observed that for both dyes, a longer exposure time resulted in a higher decolorization rate Fig. 4c. This is attributed to the extended exposure of bacterial cells to the dye solution, which enhances metabolization and the use of enzymes involved in the decolorization process^64^. These enzymes can break the chromophore present in both methyl orange and aniline red dyes.

On the other hand, regarding salt concentration, the bacterium used in this study was shown to tolerate and decolorize dyes under saline conditions Fig. 4d. This finding aligns with the report by Montañez-Barragán et al.^65^, which demonstrated that *Halomonas* sp. could decolorize four different dyes in a salinity range of 2% to 10%. These halophilic bacteria can potentially be employed in wastewater or textile industry effluents, as they might exhibit higher resistance in such environments due to their adaptive *fitness*, allowing them to tolerate the interaction between the dye and high salt concentrations^66^.

**Fig. 5:**
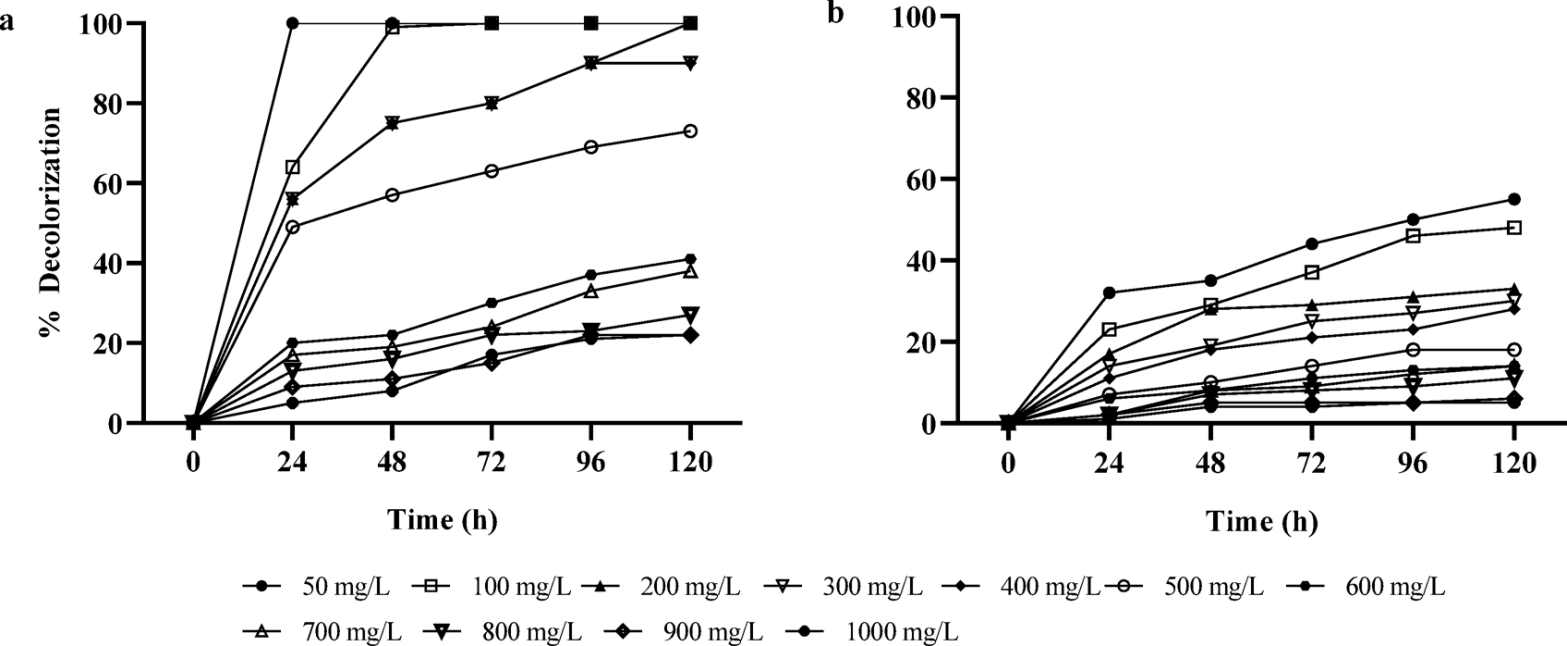
Decolorization of dye at different concentrations of *E. hirae* (Alf123) strain. (**a**) Methyl orange. (**b**) Aniline red.

After analyzing and determining the optimal parameters for the decolorization process, it was observed that the *E. hirae* (Alf123) strain demonstrated greater decolorization efficiency for methyl orange compared to aniline red (Fig. 5). At a concentration of 100 mg/L, the Alf123 strain achieved a decolorization efficiency of 50% for aniline red. However, at higher concentrations, no significant improvement in the decolorization rate was observed, and a progressive decrease in efficiency became evident. This could be attributed to the structural complexity of aniline red, which requires specific metabolic pathways for its degradation. Additionally, the accumulation of toxic intermediates from the initial breakdown of aniline red might inhibit the enzymatic activity of *E. hirae*, impairing its decolorization capacity^67^. Furthermore, the low solubility and the tendency of aniline red to form aggregates in solution could reduce its bioavailability, limiting the effectiveness of the decolorization process.

In the case of methyl orange, *E. hirae* followed a different trend, the Alf123 strain was able to completely decolorize methyl orange at concentrations of up to 200 mg/L. At concentrations up to 500 mg/L, the decolorization efficiency ranged between 90 and 60%. Beyond this concentration, the decolorization rate decreased, falling below 50% (Fig. 3). This reduction in efficiency could be related to the toxic effects of high dye concentrations on bacterial metabolic activity, possibly due to the accumulation of intermediate compounds generated during the degradation process^9^. Additionally, elevated dye concentrations may interfere with the availability of essential nutrients and disrupt the cellular osmotic balance, further compromising the efficiency of the decolorization process.

The limited ability of *E. hirae* to maintain high decolorization efficiency at elevated dye concentrations for both aniline red and methyl orange could be attributed to the structural complexity and toxicity of aniline red and its intermediates^20^. For methyl orange, high concentrations could induce metabolic stress and osmotic imbalances in *E. hirae*. To address these limitations, future studies should focus on optimizing environmental conditions, improving the bacterial strain through adaptive evolution, domestication, or genetic engineering, and evaluating the efficacy of synergistic microbial consortia to enhance decolorization rates at higher dye concentrations^68^. Additionally, the use of co-substrates could be explored to support bacterial growth under stress conditions, contributing to improved decolorization performance for both dyes at higher concentrations.

### Activity enzymatic: laccases, Lignin peroxidase, manganese peroxidase, and dioxygenases

**Fig. 6:**
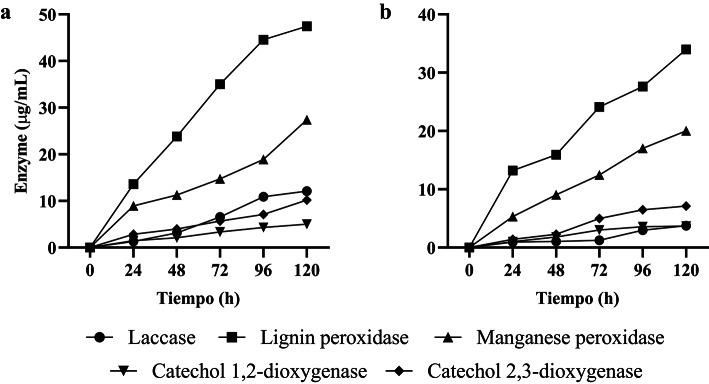
Enzymatic activity of laccases, lignin peroxidase, and manganese peroxidase**. **(**a**) Decolorization of methyl orange. (**b**) Decolorization of aniline red.

The qualitative enzymatic activity of lignin peroxidase, laccase, and manganese peroxidase was confirmed by the formation of color halos around phenolic plates. Specific color changes were observed, such as reddish-brown (guaiacol), violet (1α-naphthol), brown (2,6-DMP), and yellow (SGZ), indicating the presence of these enzymes produced by *Enterococcus hirae*. These preliminary tests were essential to validate enzyme production and justify their extraction for degradation assays of aniline red and methyl orange dyes Fig. 6. Similar qualitative tests using phenolic substrates have been reported in studies evaluating ligninolytic enzyme production^69,70^.

In the degradation assays, all enzymes reached their maximum activity on the sixth day. Previous studies have shown that enzymatic activity improves significantly after 72 h, aligning with our findings^51,71^. Among the evaluated enzymes, lignin peroxidase stood out as the most efficient, achieving a decolorization rate of 47.43% for methyl orange dye and 33.98% for aniline red dye. In second place, manganese peroxidase showed decolorization rates of 27.38% and 20.02% for methyl orange and aniline red, respectively.

This behavior can be attributed to the ability of peroxidases to act directly on the chromophores of dyes, through oxidation reactions. These reactions generate active oxygen species, such as hydroxyl radicals, which significantly contribute to the decolorization process. Peroxidases utilize hydrogen peroxide (H_2_O_2_) as a cofactor, converting it into hydroxide ions and free radicals. These reactive species interact with the dyes, decomposing the chromophores and facilitating the cleavage of azo bonds and group amine^72,73^.

However, laccase and manganese peroxidase exhibited decolorization rates below 30% for both dyes. These results may reflect inherent limitations in the use of crude enzymes, as their stability and efficiency can be affected by factors such as substrate concentration and reaction conditions. Similar findings on lower efficiency of crude enzymes have been reported in studies evaluating ligninolytic enzyme production^74,75^.

It is important to highlight that methods like enzyme immobilization could significantly enhance the efficiency of these enzymes. Immobilization can improve enzyme stability and reusability, facilitating more effective and sustainable decolorization processes^76^. This represents an opportunity to optimize decolorization rates and enable in situ applications in industrial effluent treatments. Although the evaluated enzymes demonstrated decolorization activity, especially lignin peroxidase, the results suggest the need to explore complementary strategies, such as immobilization, to enhance their efficiency in dye degradation. The findings indicate that lignin peroxidase exhibits the highest decolorization efficiency among the tested enzymes, particularly for methyl orange dye. The potential of enzyme immobilization to improve decolorization efficiency and stability presents a promising avenue for future research and application in the treatment of dye-contaminated effluents.

### Analysis of degradation products (FT-IR) and pathway of degradation

**Fig. 7:**
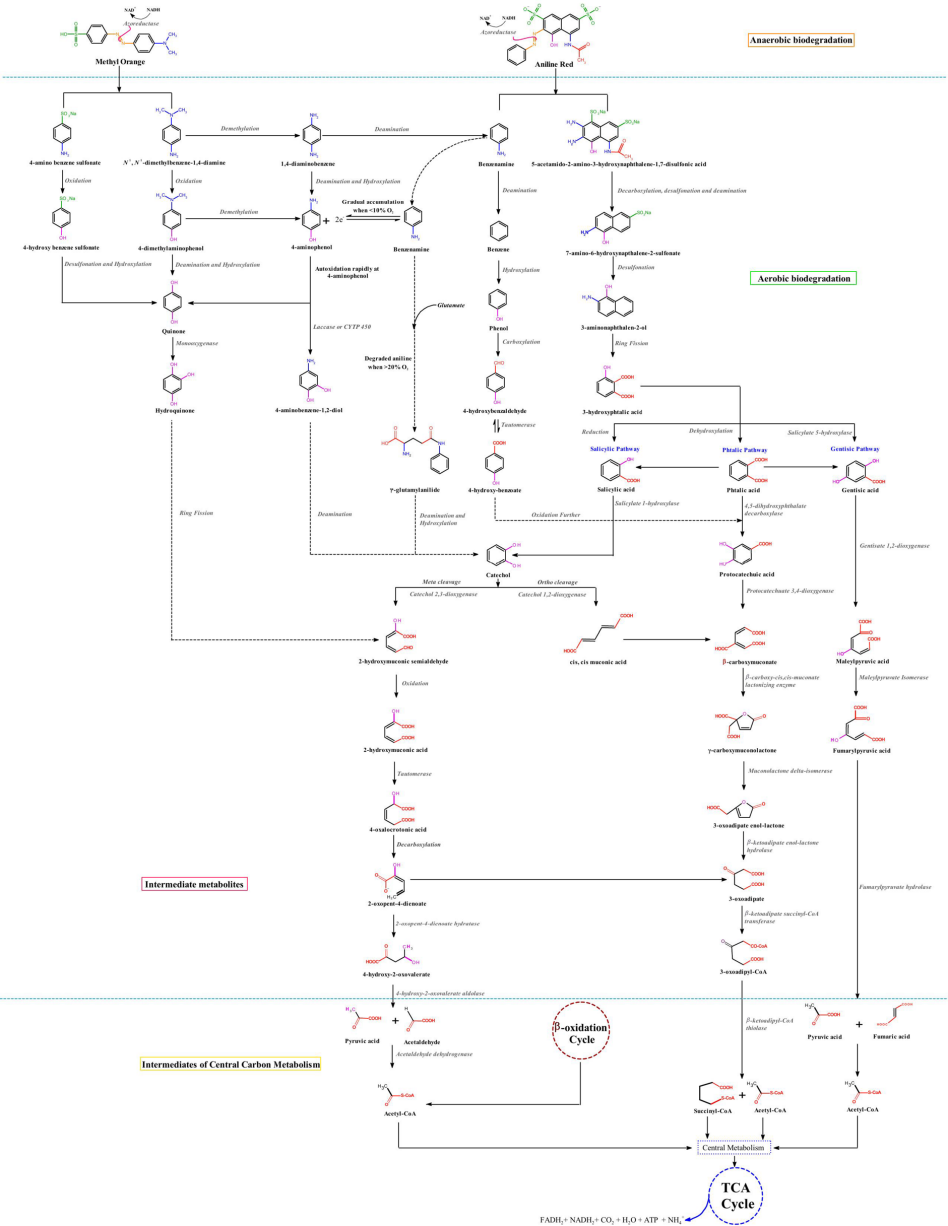
Proposed degradation pathway of methyl orange and aniline red based on spectroscopic analysis and metabolite identification.

The Fourier-transform infrared spectra of untreated and treated aniline red and methyl orange dyes are reported in the **supplementary data S1**. Based on the spectral signals obtained for the original dye and dye metabolites formed after the treatment with *E. hirae* Alf123 Table 2, we describe the structures of metabolites produced by the bacteria and discuss possible ways of their formation Fig. 7.

**Table 2:** Comparison of the FT-IR spectrum of the original dyes with metabolic products after the decolorization process by the *E. hirae* Alf123.

Degradation of Methyl orange and Aniline red				
**Step**	**Wavenumber (cm**^**-1**^**)**	**Before degradation:** **(original dye)**	**After treatment:** **Degraded metabolites**	**Observations**
	3350.22	Stretching vibrations N—H of the amine, present	Shifting peak 3350.22 to 3342.95	Represent complete degradation of azo group and change of N- H stretching to O–H stretching Formation of **tertiary aromatic amines** and **sulphonamide**
	2899.30	-C-H stretching vibration on the benzene ring	Shifting peak 2899.30 to 2961.88	
	1603.57	Absorption peak present, represent azo bond (N = N)	Disappeared, indicanting, chromophore rupture NH bend Formation of primary aromatic amines	
	1232.98	C-N bonds, Absorption peak present	Disappeared, confirming degradation of azo dye	
	1365.54	-CH_3_ stretching Absorption peak present	Formation of tertiary Aromatic amines Shifting to 1386.13	Degradation of tertiary aromatic amine: Formation of 4-dimethylaminophenol
	1473.64 and 1586.89	Presence of aromatic amino compound Absorption peaks present	Formation of aromatic primary amines Shifting to 1473.41 and 1578.18	
	1342.12	C-N vibrations in aromatic primary amine	C-N vibrations in aromatic primary amine, present peak Shifting to 1342.65	
	1031.10 1005.41 953.70	Disubstituted benzene ring	Vibrations in disubstituted aromatic ring Present peak Shifting to 1039.64 1050.01 and 953.95	Degradation of sulphonamide: Formation of quinol
	1112.24	Vibration Stretching of the S═O bond of the sulfonic group	Specific peak for the functional group: sulfite Present peak Shifting to 1112.23	
	3472.32	Group -OH substituent aromatic	-OH vibrations in aromatic ring, present peak Shifting to 3207.02	
	**Degradation of catechol as intermediate of Methyl orange** **(See point** **,** **and** **)**			Degradation and mineralization of catechol
	1147.50	Absent peak	New peak present for C–OH bond of quinol	Formation of Hydroquinone
	1580.06	Absent peak	Bend of -NH of primary amine Present peak	Formation of aniline
	1391.23	Presence of -C–C- Stretch in aromatic ring	-C–C- Stretch in aromatic ring Shifting to 1404.02	
	**Degradation of aniline ( benzenamine) as the main intermediate of Methyl orange (See point** **)**			Mineralization at CO_2_, H_2_O
	2400–2300	Absence peak	CO_2_ Absence peak	Presence of peak evidence of the mineralization
	1300–1200	Stretching vibration -C–O–C	Disappeared, confirming further degradation of intermediate metabolites	Further degradation of intermediate
**Degradation of aniline red**				
	3369.66	Stretching vibrations N—H of the amine, present	Shifting peak 3369. 66 to 3372.18	Represent complete degradation of azo group and change of N- H stretching to O–H stretching Formation of primary aromatic amines
	2962.45	-C-H stretching vibration on the benzene ring	Shifting peak 2962.45 to 2932.49	
	1632.18	Absorption peak present, represent azo bond (N = N)	Disappeared, indicanting, chromophore rupture NH bend Formation of primary aromatic amines	
	1238.10	C-N bonds, Absorption peak present	Disappeared, confirming degradation of azo dye	
	1473.27 and 1533.12	Presence of aromatic nitro compound Absorption peaks present	Retained Absorption peak present Shifting peak 1473.24 and 1538.94	Formation of benzeneamine (Primary aromatic amine)
	1343.42	Absent peak C-N vibrations in aromatic primary amine	C-N vibrations in aromatic primary amine, present peak	
	812.66- 763.88	Peaks corresponding to N–H groups attached to the benzene ring	Shifting peak 812.66 to 813.07- 763.88 to 765.17 Absorption peak present	
	1442.48	C═C stretch Present peak, Naphthalene derivatives	Retained. 1442.50 Present peak, Naphthalene derivatives	Presence of sulfonate groups in the original dye and their transformation in the treated dye
	763.88 848.41 982.54 1112.01	C-H deformation Attributed to sulphonates joins at naphthalene rings Present peak	Retained peaks Attributed to sulphonates joins at naphthalene rings	
	1442.48	Benzene ring skeleton vibration Present peak	Conserved peak Minimal increase in the intensity of absorption peak (1442.50)	A minimal decrease in the intensity of the peak at 1442.48 implied that the benzene ring was not opened by the microorganism during the degradation
	1707.89	Stretch -C═C-aromatic Present peak	Shifting to 1715.03	
	1233.08	Absence peak	Represents the C-O stretch band of phenol Present peak	Presence of species aromatics with substituents carboxylic, hydroxyls
	581.13	Deformation of the C―H bond of the aromatic ring, Absent peak	Deformation of the C―H bond of the aromatic ring Present peak	Cleavage aromatic ring of phenol possibly by action enzymatic of Laccases due to scission of the -C═C–OH, generating opening of the ring with formation of simpler compounds such as benzoic acid, carboxylic acids, carbonyls, aldehydes or saturated aliphatic
	609.57	Scission of the -C═C–OH Decrease significantly of the intensity of the absorption peaks in the region	Shifted from 609.57 to 618.57	
	1707.89	Stretching vibration of the carbonyl group Present peak	Shifted to 1715.03 Stretching vibration of the carbonyl group on the quinone ring Formation of carbonyls, carboxylic acids, aldehydes, saturated aliphatic	
	1494.01	Absorption naphthalene ring	Absent peak	Presence of benzene and naphthalene rings in original dye and treated sample
	763.88 848.41 982.54 1112.01	C-H deformation that supports unsubstituted or multisubstituted benzene and naphthalene rings Present peaks	Shifting to 765.17, 848.85, 984.63, and 1112.36	
	1385.47	Stretching of the S═O bond of the sulfonic group	Specific peak for the functional group: sulfite (S = O stretch) Shifted to 1386.20	Presence of naphthalene sulfonates in the original dye and desulphonation in the treated sample
	676.17	Sulfonated C-S bond Present peak	Disappeared	
	2179.01 2106.9	NH_3_^+^ vibrations of charged amine derivatives Present peak	Disappeared	Deamination of naphthalene Formation of phthalic derivatives
	1715.03	-COOH stretch of carboxylic acid Absence peak	Present peak	
	3486,20	group -OH substituent aromatic-	Conserved peak	Presence of 3-hydroxy phthalic structures and formation of salicylic acid by action of the hydroxyphthalate decarboxylase enzyme
	3217.70	-C–OH aromatic Present peak	Conserved peak	
	3056.22	C-H aromatic Present peak	Conserved peak	
	1751.02	O-C = O Present peak	Conserved prominent peak, indicating the presence of the carboxylic group Shifting to 1751.06	
	1666.5	O-C = O Absent peak	Conserved peak of minor intensity indicating the presence of a second carboxylic group at the benzoate ring	
	3486.20	Group -OH substituent aromatic- Present peak	Conserved peak Shifting to 3486.04	Formation of catechol
	3056.22	C-H aromatic Present peak	Conserved peak Shifting to 3056.96	
	1240.05 and 1518.15	-O–H bending vibrations of catechols and other organic alcohols Present peaks	Present peak Shifting to 1240.07 and 1518.50	
	1390.01	-O–H bending vibrations of catechols and other organic alcohols intermediates Absent peak	Present peak Shifting to 1390.04	
	1751.02	O-C = O Present peak	Shifting to 1751.06	Products intermediates of four at six carbons (unsaturated dicarboxylic acids) obtained by action of dioxygenases on aromatic ring Products intermediates substrates of TCA
	1666.5	-C = C- Absence peak	New peak	
	2932.49	-C–C- Absence peak	New peak	
	2850.03	Stretching CH aldehyde -COH Absence peak	New peak	
	1764.8	Stretching lactone Absence peak	New peak	
	1490.05	C = COO from pyruvate and succinic acid group Absent peak	New peak	
	1715.03	Ketone > C = O stretching group Absence peak	New peak	
	676.36 953.99	Polynuclear aromatic rings Present peaks	Decrease aromaticity Absence peaks	Complete cleavage of aromatic rings into aliphatic compounds leading to mineralization of aromatic rings of azo dyes
	1215.01	Aliphatic stretching -C-H Absent peak	Generation of alkanes saturated New peak	
	2932.49	CH stretching of asymmetric alkane Absence peak	Present peak	
	2850.03	CH stretching of aldehyde Absence peak	Present peak	
	2290.02	CN stretching of saturated alkyl Absence peak	Present peak

We propose that aniline red degradation can start with the reductive cleavage of the azo bond (-N = N-) mediated by azoreductases (*AzoA*) [77] , resulted in the formation of intermediates like benzeneamine  and derivatives naphthalene sulfonates . The degradation of aniline red may follow a different pathway initially, it might undergo oxidative deamination, removing the amino group (-NH_2_) and generating benzene as an intermediate product . **Benzene** can then be hydroxylated to phenol by benzene hydroxylase. Then, the resulting **phenol** is converted into phenyl phosphate by an ATP-dependent phenyl phosphate synthase and subsequently, it is converted into 4-hydroxybenzoate through carboxylation mediated by the phenyl phosphate carboxylase. The final step involves the conversion of 4-hydroxybenzoyl-CoA into the central intermediate, benzoyl-CoA, through the activity of 4-hydroxybenzoyl-CoA reductase^78^ , which is further oxidized into compounds such as catechol or hydroquinone^79^. These derivatives are susceptible to aromatic ring cleavage^80^  by the action of oxidoreductases, such as catechol-dioxygenases, through ortho or meta cleavage resulting in simpler products like dicarboxylic acids, such as maleic acid or, in some cases, fumaric acid , which are eventually transformed into CO_2_ and water in the pathway TCA^81^. Similar observations were also reported in earlier studies^27,82^.

On the other hand, the degradation of derivatives naphthalene sulfonates possibly is initiated by desulphonation and deamination of the compound **5-acetamido-2-amino-3-hydroxynaphthalene-1,7-disulfonic acid** to yield **sodium 7-amino-6-hydroxynaphthalene-2-sulfonate** **.** Next the naphthalene degradation is initiated by the hydroxylation of the aromatic rings, marking the region to be cleaved and subsequent break up of the ring. The presence of substituents groups in the 2-position like SO_3_ generates unstable intermediates, from which sulfite is eliminated spontaneously due to the rupture of the carbon sulfide (C-S) bond labile (Second desulphonation)^83^. The generated sulfite can be used as a carbon and energy source for growth of the bacteria . Concomitantly occurs the formation of 3**-amino naphthalen-2-ol.** It undergoes deamination due to the effect of rapid auto oxidation producing cleavage from the naphthalene nucleus, with formation of **3-hydroxyphthalic acid,** which after dehydroxylation converts into phthalic acid by action of the **enzyme** . The enzyme hydroxyphthalate decarboxylase acts directly causing decarboxylation and it is converted to **salicylic acid**  (Considered to be a major intermediate of naphthalene metabolism, upper pathway), which is reduced to **catechol** ^84^. However, the generated salicylate is further metabolized either via the catechol route using salicylate 1-hydroxylase (S1H) or the gentisate route employing salicylate 5-hydroxylase (S5H), which could produce Gentisic acid, Maleyl pyruvic acid, Fumaric acid and Pyruvic acid. The pyruvic acid is recognized as the first C3 compound derived from naphthalene carbon skeleton. Catechol can be presumably converted to **cis, cis-Muconic acid** and **2-Hydroxymuconic semialdehyde** by action of catechol 1,2-dioxygenase and catechol 2,3-dioxygenase enzymes, respectively, which gets cleaved to succinic acid and pyruvic acid^85^ .

These three pathways salicylate, phthalic acid and gentisate^86^ are recognized as the routes mains for the degradation of naphthalene and their derivatives. Several studies report that the bacteria could employ one, two or all pathways at same time^84,87^. The selection and use of one or other pathways depends on the organism and its genetic makeup^84^. The intermediate products obtained by these pathways due to ring fission and their entry as substrates in a TCA cycle could be mineralized to carbon dioxide and water (Lower pathway, salicylate to TCA)^88^.

We can infer that the route of salicylate to gentisate employed (S5H), could be the via favored for *E. hirae* Alf 123, such as has been reported for other microorganisms gram positives as *Rhodococcus, Micrococcus, Lachnospiraceae, Desulfotomaculum*^20,89,90^*.* A difference of Gram negative members (*Pseudomonas, Proteus*) that prefer salicylate for decarboxylation by salicylate 1-hydroxylase to yield catechol^91^.

Nevertheless, we assumed that *E. hirae* Alf123 is capable of degraded aniline red and their intermediate metabolites employing the route of salicylate and phthalic acid. This is corroborated in our study, by the presence of brown colonies in LB supplemented medium plates with aniline red (100 mg/L) that had been sprayed with a 0.3 M catechol solution, which indicate that the bacterium *E. hirae* Alf123 uses the enzyme aniline red oxygenase as the first step of degradation. According to Liang et al.^92^, these brown colonies are indicative of the accumulation and autooxidation of catechol formed from aniline red, from bacteria containing the aniline red dioxygenase (AD) gene cluster.

In this paper, we design experiments to understand pathways involved in the catechol degradation, based on the reaction products. Catechol can be degraded via either of two routes: *meta* cleavage pathway or *ortho* cleavage pathway. A visual assay by bringing the supernatant of an overnight culture to a concentration of 0.1 M of catechol. The change of color from pale yellow of LB to bright yellow was indicative that catechol was breaking down into 2 HMSA by the 2,3-dioxygenase enzyme^93^.

**Fig. 8:**
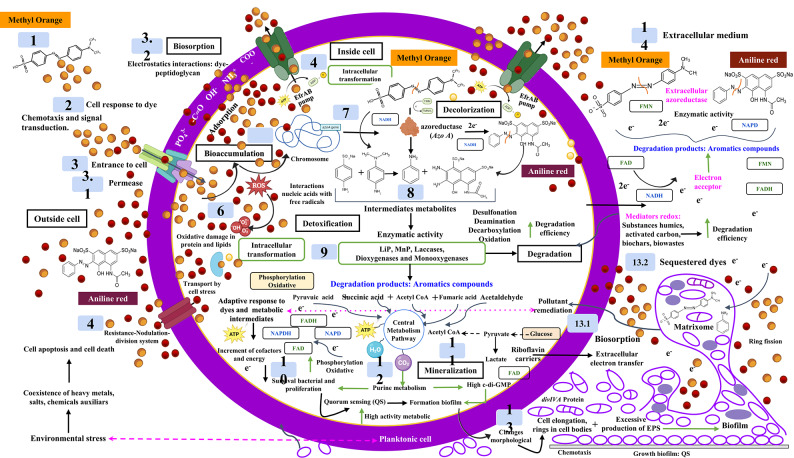
Proposed mechanism of azo dye degradation by *Enterococcus hirae* Alf123: cellular responses and metabolic pathways. Illustrated by Rosa Baldiris.

Absence of a precipitate yellow in the reaction, evidencing the *ortho* ring cleavage of catechol, by action of catechol-1,2-dioxygenase enzyme during microaerophilic degradation experiments indicating the formation of cis, cis-muconic acid. A band detected at UV 260 nm confirmed their formation Fig. 8. Moreover the 2-HMSA, characteristic of the *meta* route, was not detected neither by UV 375 nm nor by the presence of the precipitate Yellow, indicating it was absent in the reaction.

These results suggest that catechol is cleaved between the two ring hydroxyls by the enzyme, produce the compound cis, cis, muconic, which is then further metabolized in three steps to the intermediate 3-oxoadipate, which is a **convergent point** of the two branches of the $\beta$- Ketoadipate pathway. One pathway of the branches is the 3-oxoadipate and the other is the Protocatechuate. $\beta$- Ketoadipate then is converted to succinic acid, pyruvic acid and acetaldehyde, which subsequently are converted at acetyl-CoA units, incorporated into the TCA to finally produce CO_2_, H_2_O, NH_3_ and biomass^94^.

At the same time, acetyl-CoA units accelerate the TCA cycle and produce large amounts of reduction equivalents and further promote the electron transport chain to produce large numbers of electrons in the extracellular environment^95^. The azo dye being an electron acceptor accepted electrons resulting in its degradation.$\beta$-oxidation also play an essential role in the degradation of azo dyes, provide breaks the azo bond resulting in azo dye decoloration by FMN-dependent NADH azo reductase^96^.

These changes are supported by the FTIR spectral signals obtained, which indicate the formation of CO_2_ as a product of the mineralization of dye. Likewise, the liberation of NH_4_^+^ ion was evaluated in our experiment, in which *E. hirae* Alf123 was exposed to various treatments such as catechol, naphthalene and salicylate, showing the ability of *E. hirae* Alf123 to use them as the sole source of carbon and nitrogen. During this assay *E. hirae* showed their capacity of degraded azo naphthalene sulfonates dyes and other aromatics compounds.

Based on the results obtained in this study, we assumed that aniline red is metabolized via catechol as the first intermediate under aerobic conditions, and that 4-aminobenzoic acid is the first intermediate produced under anaerobic conditions by reductive deamination of 4-aminobenzoyl- CoA. These results are like a report by Kumari et al.^97^ and Schnell and Schink^98^. However, additional studies are required to establish it is *E. hirae* using the gentisate route.

**For methyl orange**, degradation can start with the reductive cleavage of the azo bond (-N = N-) mediated by azoreductases (AzoA)^77^**,** two intermediates can be generated like N,N′-dimethylbenzene-1,4-diamine and 4-aminobenzene-1-sulfonic acid . N,N′-dimethylbenzene-1,4-diamine further undergo deamination and convert 4-dimethyl aminophenol, while 4-aminobenzene-1-sulfonic acid by action of the 4-sulfophenyl monooxygenase enzyme is convert to sulfonamide with change of the amino group to hydroxyl (OH-) group, then undergo oxidation leading to the formation of the compound **4-hydroxybenzene sulfonate**, which is attack by oxygenases, undergoes desulphonation and hydroxylation producing quinol . At the same time 4-dimethylaminophenol also can be converted to a **quinol** by oxidation of the tertiary amine. Next, quinol is hydroxylated by a flavoprotein monooxygenase for produce **hydroquinone** . As degradation progresses, undergoes further dehydroxylation, generanting **catechol** , a change of position of the hydroxyls from *para* position to *ortho* position is generated, which further cleaves to produce **cis, cis muconic acid or 2-Hydroxymuconic semialdehyde** by action of catechol 1,2-dioxygenase and catechol 2,3-dioxygenase enzymes, respectively . Subsequently the intermediates products generated are integrated as substrates to central carbon mechanism  and finally mineralized to CO_2_ and H_2_O .

However, N,N′-dimethylbenzene-1,4-diamine further can go the other route and formed benzeneamine as product of the demethylation and desamination. The benzeneamine is considerate as the main intermediate in the degradation of many azo dyes , these compounds would follow the same route of degradation described for benzenamine and aniline red dye **.**

The proposed mechanism and its metabolic pathways for the degradation of methyl orange and aniline red by *E. hirae* Alf123 were constructed based on the results obtained from our study of decolorization assays, enzyme activity, UV–vis, FT-IR spectroscopy and literature review. Figure 8 depicts a schematic illustration of the proposed mechanism.

### Mechanism proposed degradation of methyl orange and aniline red 1 by *E. hirae Alf123*

*E. hirae* Alf123 showed the ability to decolorize methyl orange and aniline red suggesting they have different mechanisms to adapt and detoxify the presence of azo dyes in its environment.

Our findings suggest that the process begins when the bacteria detect the presence of azo dyes in the extracellular medium , in response to environmental stress, by chemotaxis, the interaction between ligands and respective receptors induce a conformational change to generate signal transduction . The first molecule in the chemotaxis pathway, activates chemotaxis ligands through the chemokine receptors on the cell surface . Once recognized, the dye allows it to enter the bacterium in two ways, either by permeating the plasma membrane using transport proteins (permeases) due to the high concentration found in the extracellular space or by biosorption , having electrostatic interactions with bacterial peptidoglycan of the cell wall, facilitating its captake and possible temporary retention.

Among these mechanisms are the use of specific transporters and proteins, both peripheral and transmembrane, which facilitate the movement of dyes via ABC-type pumps or resistance-nodulation-division systems ^99^. These systems actively expel toxic dyes from the cell, thereby protecting the bacterium and maintaining its *fitness* to modulate the concentration and/or presence of the dyes.

Bioaccumulation  can also occur, when dyes are transported into the cell via active transport pumps, such as ABC transporters systems (EfrAB pump), enabling their storage or intracellular transformation^100^. These processes generate oxidative stress , where reactive oxygen species (ROS) are produced, causing damage to the essential proteins and lipids of the bacteria. Furthermore, *E. hirae* may utilize proteins associated with oxidative stress, such as catalases, superoxide dismutases, glutathione peroxidases or peroxiredoxins, which help manage ROS generated during chemical interactions with dyes, especially by superoxide anion radicals (•O^2^ −) and hydroxyl radicals (•OH)^101^. These proteins not only protect the cell from oxidative damage, but can also facilitate chemical transformation processes of dyes, enhancing their degradation. To counteract this oxidative stress, the bacterium activates defense mechanisms, including the cellular stress response and the Resistance-Nodulation-Division (RND)-like resistance system, which contributes to the expulsion of the dye, into the extracellular medium via EfrAB pump-like efflux pumps. Other mechanisms include genetic changes resulting from changes in protein sequences and structures^102^. Finally, heat shock proteins could play an indirect role by repairing proteins damaged by interactions with reactive products generated during dye degradation. Together, these protein systems, along with enzymatic and non-enzymatic mechanisms, strengthen the ability of *E. hirae* to tolerate and degrade complex aromatic compounds such as aniline and derivative naphthalene sulfonates.

As a defense mechanism against the dye, a gene encoding a protein that favors the degradation begins to be expressed; this is Azoreductase (AzoA) ^77^. AzoA is an aerobic FMN dependent NADH azoreductase present in several *Enterococcus* strains, characterized as an azoreductase of broad spectrum, which support their ability to reduce azo dyes by NADH as a preferred electron donor^103^. NADH facilitates the reduction of azo bond, transferring two electrons (2e^-^) at the azoreductase, which catalyzes the cleavage of the Azo bond (-N = N-), leading to discoloration. Their quinone reductase activity.

As a result of this catalysis of the azo bond, different products are generated that depend on the structure of the dye. In the case of Methyl Orange two intermediates can be generated like N,N′-dimethylbenzene-1,4-diamine and 4-aminobenzene-1-sulfonic acid . For aniline red, the benzenamine and 5-acetamido-2-amino-3-hydroxynaphthalene-1,7-disulfonic acid are formed . Alongside azo reductases, the degradation of methyl orange and aniline red dyes may involve many enzymatic pathways catalyzed by other enzymes, such as lignin peroxidase (LiP), manganese peroxidase (MnP), laccases, monooxygenases and dioxygenases , which are useful to the detoxification and its conversion into biodegradable compounds^80^.

Under aerobic conditions, considering that this bacterium is facultative, aromatic compounds generated such as benzene, phenols, hydroquinone, catechol and protocatechuate need to be converted to metabolites from the central metabolic pathways to achieve full assimilation by bacteria . Depending on the type of aromatic derivative, the ring cleavage is made in specific sites which result in diverse products (Fig. 8).

Aniline (benzenamine), as the main intermediate of the degradation of methyl orange and other dyes can be degraded depending on oxygen availability, aniline can follow different metabolic pathways: in low oxygen conditions (< 10%), it accumulates and can be slowly transformed into 4-aminophenol, while in high oxygen concentrations (> 20%), it is rapidly degraded by oxidation, generating products such as benzene and 4-aminophenol, and in much higher oxygen conditions aniline can be converted to catechol and this in turn by different enzymes can be processed into biodegradable intermediates such as cis-, cis-muconic acid and 2-Hydroxymuconic semialdehyde, which by action of catechol 1,2-dioxygenase and catechol 2,3-dioxygenase enzymes, respectively, are split into succinic acid, fumaric acid, acetaldehyde and pyruvic acid ^85^.

On the other hand, the degradation of aniline may follow a different pathway initially, it might undergo oxidative deamination, removing the amino group (-NH_2_) and generating benzene as an intermediate product. Benzene can then be hydroxylated to phenol, which is further oxidized into compounds such as catechol or hydroquinone^79^. These derivatives are susceptible to aromatic ring cleavage by the action of catechol-dioxygenases, resulting in simple products like dicarboxylic acids, such as maleic acid, succinic acid, pyruvic acid, acetyl CoA, or, in some cases, fumaric acid^81^. These metabolic intermediates formed generally are oxoacids that are integrated into the tricarboxylic acid (TCA) cycle, where they are completely mineralized into CO_2_, H_2_O and NH_3_ , which can be used for cellular energy production and for the production of electron-generating cofactors in the electron transport chain, as NADH_2_, FADH_2_ that promote their discoloration and degradation of the dye.

The CO_2_ generated is incorporated into purine metabolism , stimulating bacterial proliferation and activation of quorum sensing, which increases metabolic activity and promotes the transition from planktonic cells to biofilms. During this process the levels of cyclic di-guanosine monophosphate (c-di-GMP) a signaling molecule that regulates the formation of biofilms, are increased, the bacterial cells can modify their morphology  while the stages of this process are taking place, due to the overexpression of the *div*IVA protein, which generates an elongation of the cells, the ring formation in the cell body and an excessive production of exopolysaccharides (EPS)^104^. Through EPS matrices the extracellular transfer of electrons occurs by a mechanism mediated by riboflavin carriers, which carry the electrons produced inside the cells through biofilm matrix by hopping, a recognized pathway as prevailing for the extracellular electron transfer^105^. These electrons can interact with key outer membrane proteins (OsRP, OmcB, OmcC, OmcZ, OmcS, OmcE and PilA) which may act as extracellular, terminal azoreductases producing the bio decolorization of azo dyes by a process neatly extracellular. However, the process of reduction intracellular or extracellular, depends on the structural components of each bacteria^106,107^. In other words, the bio decolorization of azo dyes may proceed by a pathway independent of azoreductase. Other mechanisms of Extracellular Electron Transfer can occur used the fermentation, pyruvate is oxidized a lactate in presence of Lactate dehydrogenase, the electrons are transferred at space extracellular, where mediators redox facilitated the pathway and finally are taken by acceptors electrons extracellularly, who conducted the reduction and the growth of the biofilm^108^.

Biofilm formation also increases biosorption efficiency, allowing retention of the dye in the extracellular matrix (Matrixome) , where it is sequestered and subsequently degraded by soluble azoreductase enzymes, thus decreasing the possibilities of entering the bacteria and its toxicity. The dye absorbed also can be indirectly reduced by NADPH dependent oxidoreductase^109^. Additionally, other redox pathways are activated through the utilization of redox mediators such as phosphates, ammonium, carbonyl and hydroxyl groups, which also assist in the decolorization of the dyes and their intermediates . Finally, this process can be enhanced by using activated carbon, residues, and biochar, which facilitate electron transfer and increase the efficiency of the process, which acs as mediators redox.

Based on multiple omics datasets, a diagram elaborating on the possible degradation of dyes utilization mechanisms in *E. hirae* was drawn Fig. 8, which include metabolism of intermediate metabolites and reduction to final products used as nutrient and energy sources for the microorganism. This diagram provided a wide range of potential targets for bioremediation; however, many details still require further investigation.

Comparative analysis of the 62 genomes of *E. hirae* consulted in https://img.jgi.doe.gov, (accessed February 2025), corroborated the presence of genes associated with this mechanism. However, no key genes were identified in plasmids, such as *odt* and *tnpA*, suggesting that aniline degradation might be incomplete. In our study, this would explain why, at high aniline concentrations, the dye degradation process was not complete. A difference in Methyl orange, that was able to completely decolorize and degrade.

### Phytotoxicity analysis

The biological model *Lactuca sativa* is widely used as a tool to assess the phytotoxicity of various chemical compounds due to its sensitivity compared to other plant species. Parameters such as root and hypocotyl elongation are key indicators for determining the toxic effects during the early stages of plant development^110^.

**Fig. 9:**
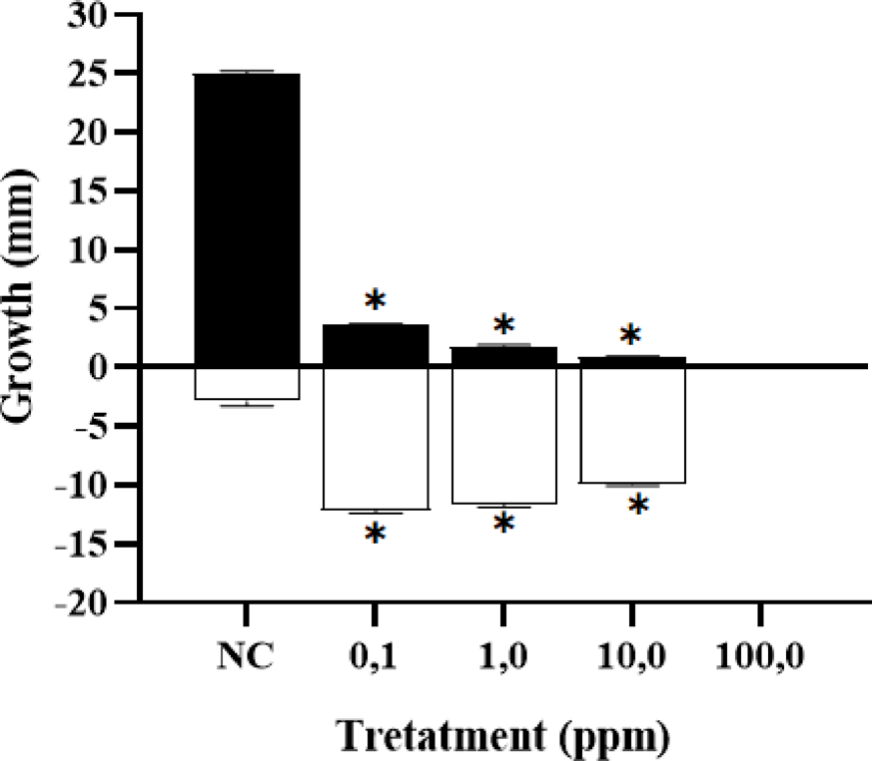
Phytotoxicity of dyes and their degradation products by *Enterococcus hirae* (Alf123).

In this study, the results obtained Fig. 9 show that the treatments presented an asymmetric distribution, as evidenced by the Shapiro–Wilk normality test (*P* = *0.0037; p* > *0.05*). Additionally, a statistically significant difference was observed between the means of the treatments with respect to root and hypocotyl growth (*P* = *0.025*). These findings reveal that the increase in the concentration of secondary metabolites resulted in greater toxicity toward *L. sativa*.

Similar patterns have been reported in previous studies, such as Aounallah et al.^111^, who demonstrated that the degradation products of the azo dye Congo red were more toxic than the original compound in plant species such as *Amaranthus mangostanus* and *Sesamum indicum*. This increase in toxicity can be explained by the generation of secondary metabolites during degradation, which may interfere with key cellular processes, such as cell division, protein synthesis, and membrane homeostasis.

Where: Black bars represent radicle growth, white bars represent hypocotyl growth, and * indicates significance at an *alpha value of 0.05*.

The toxicity observed could be attributed to the accumulation of aromatic compounds, such as substituted amines, or to the formation of products derived from the enzymatic breakdown of dyes^112^. This process may involve enzymes distinct from azoreductases, which, during dye cleavage, can generate compounds such as diazonium salts, which are highly reactive and toxic to plant tissues^113^. These results suggest that, although the dye is degraded, the intermediate and final products of the detoxification process may have significant phytotoxic effects (*P* < *0,0001*).

## Electronic supplementary material

Below is the link to the electronic supplementary material.

Supplementary Material 1

## Data Availability

The 16 s rRNA gene sequence of *Enterococcus **hirae* Alf123 strain generated in this study has been deposited in the NCBI database under the accession number PQ820966. All other datasets generated and/or analyzed during the current study are available from the corresponding author on reasonable request.
