# Supplementary material for: Decolorization of methyl orange and aniline red dyes by Enterococcus hirae isolated from beach sand
Source: Sci Rep. 2025 Sep 29;15:33341. doi: 10.1038/s41598-025-12584-5 (PMC12480510; doi:10.1038/s41598-025-12584-5)
Supplement: Supplementary file 1 — Supplementary Material 1 [file 41598_2025_12584_MOESM1_ESM.docx]

**Supplementary Material**

**Figure S1**. FT-IR spectrum Aniline Red before degradation.

**Figure S2**. FT-IR spectrum Aniline Red after degradation.

**Figure S3**. FT-IR spectrum Methyl Orange before degradation.

**Figure S4**. FT-IR spectrum Methyl Orange after degradation.
